# Association rare d'une maladie de Takayasu et d'une maladie inflammatoire chronique intestinale au Gabon

**DOI:** 10.48327/mtsi.v3i3.2023.386

**Published:** 2023-09-27

**Authors:** Josaphat IBA BA, Annick Flore MFOUMOU, Monique MBOUNJA, Léonie LÉDAGA LENTOMBO, Ulrich Davy KOMBILA, Marielle IGALA, Jean Bruno BOGUIKOUMA

**Affiliations:** 1 Service de médecine interne, Centre hospitalier universitaire de Libreville, Libreville, Gabon; 2 Service de gastro-entérologie, Centre hospitalier universitaire de Libreville

**Keywords:** Maladie de Takayasu, Maladie inflammatoire chronique intestinale, Gabon, Afrique subsaharienne, Takayasu's disease, Chronic inflammatory bowel disease, Gabon, Sub-Saharan Africa

## Abstract

RÉSUMÉ La maladie de Takayasu est une vascularite affectant les vaisseaux de gros calibre, particulièrement l'aorte et ses branches principales, pour laquelle le rôle de *Mycobacterium tuberculosis* a été évoqué comme déclencheur par une réaction d'hypersensibilité. Les maladies inflammatoires chroniques intestinales peuvent être cliniquement confondues en Afrique subsaharienne avec les maladies parasitaires. Nous rapportons une association rare de maladie de Takayasu avec une maladie inflammatoire chronique intestinale dans la population gabonaise.

## Abstract (en)

Takayasu's disease is a vasculitis affecting large vessels, particularly the aorta and its main branches, for which the role of *Mycobacterium tuberculosis* has been suggested as a trigger by a hypersensitivity reaction. Inflammatory bowel diseases, which in sub-Saharan Africa can be confused with parasitic diseases, can rarely be found in association with Takayasu's disease. We report an association between both diseases in the Gabonese population.

## Observation

**Figure 1:**
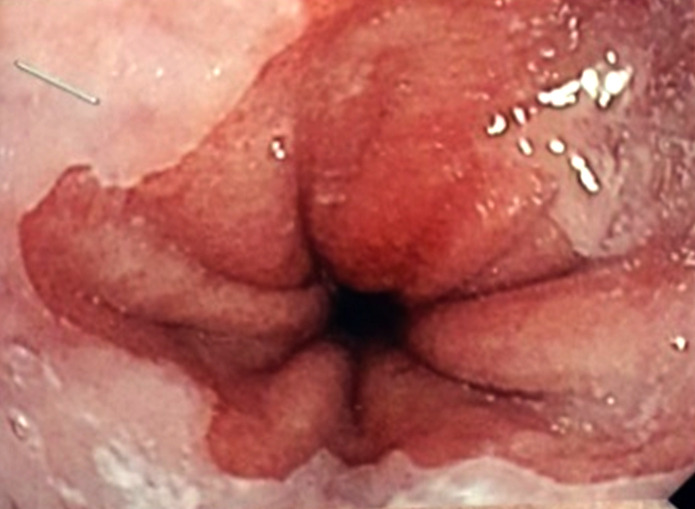
OEsophagite peptique de grade 2 Grade 2 peptic esophagitis

**Figure 2:**
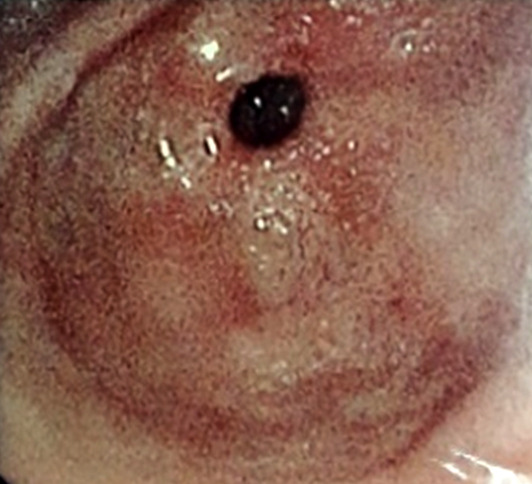
Lésions angiomateuses à 30 centimètres des arcades dentaires Angiomatous lesions 30 centimeters from dental arches

**Figure 3:**
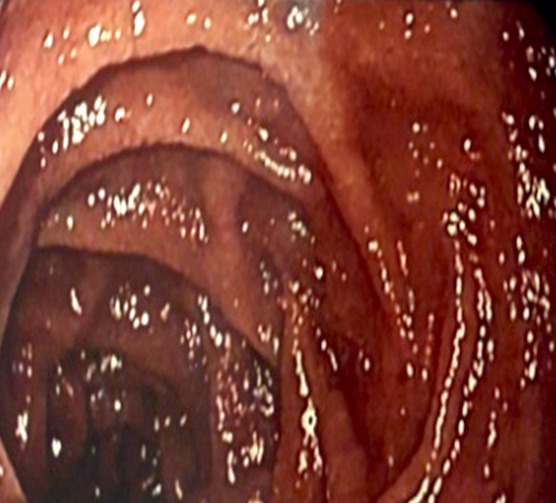
Gastrite antrale et fundique Antral and fundic gastritis

Un patient de 65 ans, informaticien, signalant des antécédents de consommation d'alcool (30 grammes/jour, sevré depuis 5 mois), de décoctions traditionnelles, et signalant une épigastralgie chronique depuis 2 ans malgré la prise d'inhibiteur de la pompe à protons, présentait en décembre 2021 une sensibilité épigastrique avec arrêt des matières sans arrêt des gaz. L'endoscopie digestive haute mettait en évidence une oesophagite peptique de grade 2 (Fig. 1), des lésions angiomateuses à 30 centimètres des arcades dentaires (Fig. 2) et une gastrite antrale et fundique érosive et pétéchiale (Fig. 3).

Les biopsies confortaient la gastrite chronique, avec recherche d’*Helicobacter pylori* négative. Le patient était traité favorablement par l'association oméprazole 40 mg/jour et lévosulpiride 75 mg/jour. Puis en février 2022, apparaissait un hoquet récurrent faisant suite à une douleur post-prandiale tardive, spasmodique paroxystique de la fosse iliaque gauche à tendance invalidante, extensive à tout l'abdomen. Cette douleur conduisait à une restriction alimentaire du patient, génératrice d'une perte de poids de 16 kg en 3 mois, qui motivait son hospitalisation dans le service de gastro-entérologie du 22 au 31 mars 2022. Les conjonctives étaient bien colorées. Le patient était apyrétique, avec un état général conservé malgré la perte de poids. Le poids était de 74 kg pour une taille de 1,72 m soit un indice de masse corporelle de 25 kg/m^2^. La pression artérielle sous traitement anti-hypertenseur était de 120/80 mmHg, la respiration eupnéique avec une saturation en oxygène à l'air ambiant à 98%. À l'examen clinique, il n'existait pas de sensibilité abdominale, d'hépatomégalie, de splénomégalie, ni d'adénopathies périphériques. L'auscultation respiratoire et cardiaque était sans particularités, et sur le plan vasculaire était retrouvée une diminution du pouls radial gauche. Le patient a été secondairement transféré dans le service de médecine interne.

Sur le plan biologique, l'hémogramme retrouvait 7 700 leucocytes/mm^3^ (6406 neutrophiles/ mm^3^, 7 238 lymphocytes/mm^3^), une anémie à 10 g/dL microcytaire, et 194 000 plaquettes/ mm^3^. La C-réactive protéine était à 28,4 mg/L et le bilan rénal (urée, créatinine) normal. La recherche sur les selles de kystes, d'oeufs, d'amibes et de parasites était négative. La sérologie bilharzienne et syphilitique (TPHA, VDRL) était négative, et la sérologie amibienne positive à un titre de 1/160 (titre ≤ 160 = réaction douteuse).

**Figure 4:**
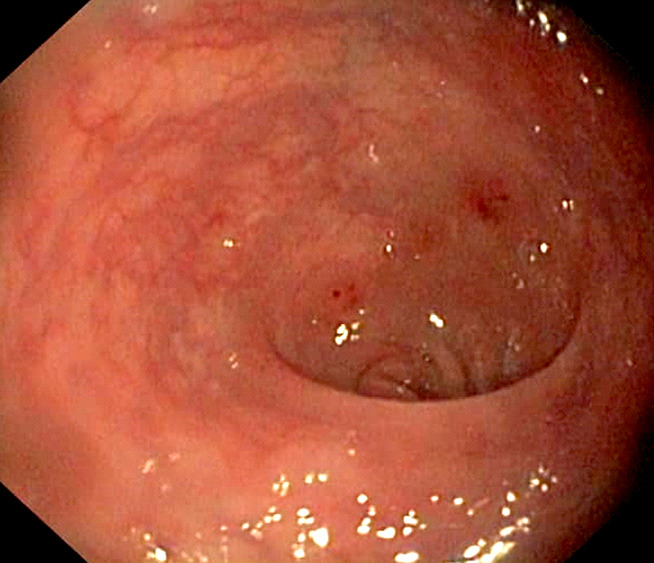
Lésions érosives avec intervalle de muqueuse saine Erosive lesions with interval of healthy mucosa

**Figure 5:**
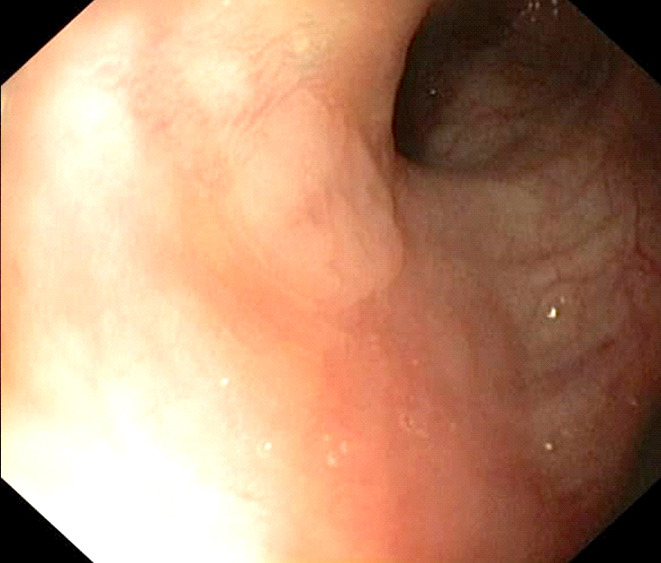
Lésion de polype plan 0-IIa (adénome simple) Plane 0-IIa polyp lesion (simple adenoma)

Sur le plan morphologique, la coloscopie retrouvait à 30 cm de la marge anale 2 à 3 lésions congestives peu hémorragiques, sans diverticule ni polype, dont l'histologie était en faveur d'une colite inflammatoire et oedémateuse. Le dosage des anticorps anti-*Saccharomyces cerevisiae* (ASCA) et des anticorps anti-cytoplasme des neutrophiles (ANCA) était non contributif. La tomodensitométrie thoraco-abdominale retrouvait des calcifications pariétales de l'artère sous-clavière gauche, de la crosse de l'aorte, de l'aorte thoracique descendante, sans anévrysme. L’échocardiographie retrouvait une fraction d’éjection à 80% sans valvulopathies. L’échodoppler des artères sous-clavières était sans particularité, et celle des membres inférieurs en faveur d'une athérosclérose des artères fémorales distales, poplitées et du réseau sous poplité, avec multiples plaques hétérogènes avec quelques-unes calcifiées intéressant les fémorales superficielles, distales, et artères tibiales postérieures bilatérales. Le diagnostic de maladie de Takayasu (MT) était établi sur les critères de Shalma *et al*. [8], par l'existence de 2 critères majeurs (atteintes pariétales de l'artère sous-clavière gauche, de la crosse de l'aorte, de l'aorte thoracique descendante documentées par angioscanner, et du pouls radial diminué au membre supérieur gauche) et de 3 critères mineurs (HTA, aorte thoracique descendante sans anévrysme, et athérosclérose étendue des membres inférieurs), stadifiant la maladie de Takayasu en type II-b. Elle était associée à une colite inflammatoire et oedémateuse (Fig. 4) avec présence de 3 lésions polyploïdes (Fig. 5 et 6). Le hoquet était traité favorablement par de la chlorpromazine 25 mg injectable (1/2 ampoule x2/jour). Le patient a été traité pour la MT par une corticothérapie de 1 mg/kg/jour soit 70 mg/jour couplée à un immunosuppresseur pour lequel notre choix allait vers l'azathioprine (Imurel) à raison de 150 mg/jour, à laquelle étaient associés concernant la colite inflammatoire la mésalazine 4 g/jour et le métronidazole 1500 mg/jour permettant la disparition des douleurs spasmodiques et une reprise pondérale progressive.

**Figure 6:**
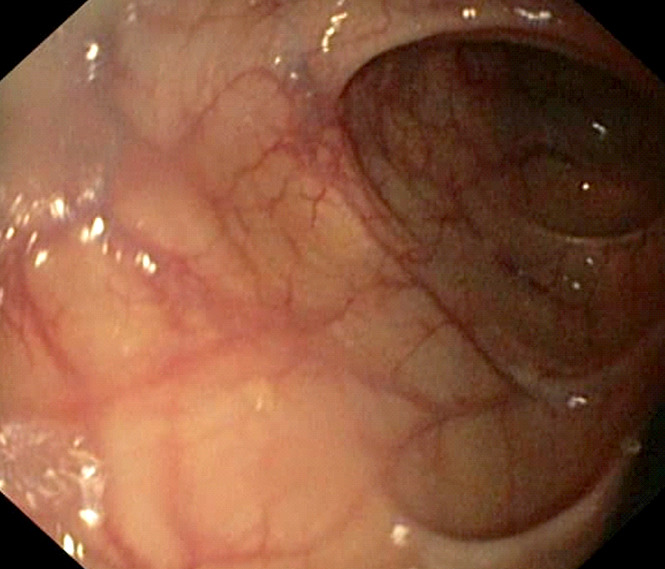
Polypes sessiles 0-IS (lésion festonnée sessile) Sessile polyps 0-IS (sessile scalloped lesion)

## Discussion

L'association maladie de Takayasu (MT) et maladie inflammatoire chronique intestinale (MICI) a été décrite pour la première fois avec la maladie de Crohn en 1976 par Yassinger *et al*. [9]. Si l'association de la MT avec la maladie de Crohn semble plus courante, celle avec la rectocolite hémorragique (RCH) demeure exceptionnelle. Ces deux affections qui sont des maladies inflammatoires chroniques ont une étiologie inconnue, mais seraient sous la dépendance de facteurs génétiques (qui déterminent une susceptibilité à développer ces maladies) et environnementaux (véritables déclencheurs des symptômes), auxquels on ajoute le microbiote intestinal. Elles évoluent par des phases de poussées-rémissions et partagent une pathogenèse commune. Il a été en effet suggéré qu'il pourrait coexister une réaction croisée entre les auto-antigènes de la paroi artérielle et ceux de la muqueuse colique. Le TNFα qui a été identifié dans la paroi vasculaire de la vascularite des gros vaisseaux [3] serait, en association avec d'autres cytokines pro-inflammatoires (interleukines 6, 8, 12 et 18), responsable d'une amplification de la prolifération artérielle intimale conduisant sur le plan évolutif à la constitution de sténose endoluminale [7], et sur le plan anatomopathologique à la constitution de lésions histopathologiques granulomateuses également retrouvées au cours des MICI.

Esatoglu *et al*. dans une série de 238 MT retrouvaient l'association avec une MICI en seconde position après l'association avec la spondylarthrite ankylosante, soit respectivement 6 et 8% [1]. Notre observation constitue la première association MT-MICI rapportée dans la revue de la littérature d'Afrique subsaharienne. Dans cette association, nous avons concernant la MICI exclu : a) les colites parasitaires qui peuvent simuler des MICI, avec au premier plan les schistosomiases (sérologie négative dans notre observation) et amibiases (taux non significatif chez notre patient). Iba Ba *et al*. [4], dans une série de 4 rectocolites hémorragiques (RCH) documentées au Gabon, avaient retrouvé la coexistence de la MICI avec une amibiase dans un cas, et une association amibiase et bilharziose (affections sévissant à l’état d'endémie dans ce pays) dans un second cas, qui pourrait participer à amplifier la réaction inflammatoire hémorragique colique; et b) les colites d’étiologie tumorale par biopsies. Dans tous les cas, nous pouvons dans cette observation retenir le diagnostic d'une colite inflammatoire indifférenciée associée à la maladie de Takayasu.

Les anticorps anti-*Saccharomyces cerevisiae* (ASCA) et anti-cytoplasme des neutrophiles (ANCA) sont utilisés comme marqueurs sérologiques des MICI. Les ASCA sont rapportés chez 50 à 80% des patients atteints de maladie de Crohn et 2 à 14% des patients atteints de RCH, alors que les ANCA (surtout p-ANCA) sont retrouvés chez 40 à 80% des patients atteints de RCH et 5 à 25% des patients atteints de maladie de Crohn. Ces anticorps anti-*Saccharomyces cerevisiae* (ASCA) et anticytoplasme des neutrophiles (ANCA) ne sont pas dans la revue de la littérature significativement augmentés en cas d'association avec la MT. On les retrouve positifs pour Ozbakir *et al*. [5] (dans une série de 32 associations MT-MICI) et Reny *et al*. [6] (dans une série de 44 patients avec MICI) dans seulement 9% des cas.

Sur le plan thérapeutique, notre patient a été traité comme rapporté dans la littérature par l'association de corticothérapie 1 mg/kg/jour soit 70 mg/jour couplée à un traitement immunosuppresseur dans un but d’épargne cortisonique pour lequel notre choix s'est orienté vers l'azathioprine 150 mg/jour. Des thérapies biologiques comme les anti-TNF ou le tocilizumab peuvent être utilisées dans les maladies réfractaires ou récurrentes [2].

## Conclusion

MICI et MT partagent des données physiopathologiques communes autorisant la possibilité de traitements communs. Leur association demeure rare et méconnue par les praticiens d'Afrique subsaharienne.

## Contribution Des Auteurs

Josaphat IBA BA, conception, rédaction, relecture; Annick MFOUMOU, rédaction et relecture; Monique MBOUNJA, rédaction et relecture; Léonie LÉDAGA LENTOMBO, recherche biblio-graphique; Ulrich Davy KOMBILA, relecture; Marielle IGALA, relecture; Jean Bruno BO-GUIKOUMA, conception, relecture et approbation de la version finale.

## Liens D'intérêts

Les auteurs déclarent n'avoir aucun conflit d'intérêts.
